# Digitale Public Health – Welche Voraussetzungen sind notwendig, um Versprechen und Potenziale einzulösen?

**DOI:** 10.1007/s00103-024-03839-z

**Published:** 2024-03-11

**Authors:** Hajo Zeeb, Iris Pigeot, Benjamin Schüz

**Affiliations:** 1 Leibniz-Institut für Präventionsforschung und Epidemiologie, Bremen – BIPS, Achterstr. 30, 28359 Bremen, Deutschland; 2 Fachbereich Human- und Gesundheitswissenschaften, Institut für Public Health und Pflegeforschung, Universität Bremen, Grazer Straße 4, 28359 Bremen, Deutschland; 3 Health Sciences Bremen, Universität Bremen, 28359 Bremen, Deutschland; 4 Fachbereich Mathematik und Informatik, Universität Bremen, 28359 Bremen, Deutschland

Das erste Kapitel der „Globalen Strategie zu digitaler Gesundheit 2020–2025“ der WHO mit dem Titel „Digitale Technologien – die Zukunft der globalen Gesundheit gestalten“ zeigt, dass mit der zunehmenden Digitalisierung und den Möglichkeiten des digitalen Informations- und Datenaustauschs große Erwartungen verknüpft werden. Diese erstrecken sich nicht nur auf das Gesundheitswesen an sich, sondern auch auf Prävention, Gesundheitsförderung und Gesundheitspolitik bis weit hinein in die UN-Nachhaltigkeitsziele. Die COVID-19-Pandemie hat hier an vielen Stellen als Triebfeder gewirkt. Dies hat auch das „European Centre for Disease Prevention and Control“ (ECDC) im Rahmen einer Expertenkonsultation festgehalten und klar herausgestellt, dass sich der Einsatz von digitalen Technologien für Public-Health-Schlüsselfunktionen an der Erreichung von Public-Health-Zielen messen lassen muss. Der Bericht zu dieser Konsultation, der während der Pandemie Mitte 2021 erschien, stellt außerdem die Bedeutung von Standards, Interoperabilität und Governance sowie von Partizipation und Co-Design u. a. zur Vertrauensentwicklung der Bevölkerung in diese Technologien heraus. Und nicht zuletzt werden robuste und umfassende Monitorierung und Evaluation gefordert, damit beurteilt werden kann, ob Public-Health-Funktionen umgesetzt und Ziele erreicht werden.

Wenn man die COVID-19-Pandemie als ungeplanten, herausfordernden Anwendungsfall für die Digitalisierung von Public Health betrachtet, dann wird schnell klar, dass Erfolge und Misserfolge, Bleibendes und Passageres oft eng beieinander lagen. Der extrem intensivierte, datengetriebene Informationsfluss zu vielen Aspekten der Pandemie wurde zum Teil durch eine schwer differenzierbare Gemengelage von Falsch- und Fehlinformationen konterkariert. Und dass Digitalisierung sozial benachteiligte Bevölkerungsgruppen zurücklassen kann, zeigte sich im Rahmen der Pandemie ebenfalls. Deutschland und viele andere Länder haben dadurch eine Art Crashkurs in Digital Public Health durchlaufen. Was sich daraus als nachhaltig erweist und wie neue Strategien die während der Pandemie generierten Erkenntnisse aufnehmen, ist noch Gegenstand der wissenschaftlichen und politischen Diskussion. Mit den Möglichkeiten der künstlichen Intelligenz ist ein weiterer Beschleunigungsfaktor im Themenfeld digitaler Gesundheitstechnologien dazugekommen, der das interdisziplinäre Wissenschafts- und Praxisspektrum von Public Health sowohl in Anwendung als auch in Lehre und Forschung weiterentwickeln kann.

Die Beiträge des vorliegenden Heftes sind überwiegend im Kontext der Arbeit des Leibniz WissenschaftsCampus Digital Public Health Bremen (LWC DiPH) 2020–2023 entstanden. Der einführende Beitrag von *Zeeb und Koautor:innen* blickt auf die COVID-19-Pandemie zurück, die quasi zeitgleich mit dem LWC DiPH begann und mit hoher Dynamik Digitalisierungsthemen in Public Health brachte. Dies betrifft epidemiologisches Monitoring und Erhebungsmethoden ebenso wie Arbeitsweisen und Strukturen, zum Beispiel im öffentlichen Gesundheitsdienst.

Nach dieser allgemeinen Einführung setzen sich die nächsten 3 Beiträge mit sozialen und rechtlichen Rahmenbedingungen auseinander. *Brand und Koautorinnen* zeigen mittels eines narrativen Reviews und eigener empirischer Ergebnisse zur digitalen Spaltung (Digital Divide), dass zur bedarfsgerechten Entwicklung von digitalen Technologien für Public Health sozial benachteiligte Adressatengruppen stärker eingebunden werden müssten.

*Dratva und Koautor:innen* liefern eine kritische Betrachtung zu Konzepten und Stand der digitalen Gesundheitskompetenz (DGK) in Deutschland. Empirische Studien zeigen trotz leichter Zugewinne während der COVID-19-Pandemie erhebliche DGK-Defizite in der Bevölkerung auf, insbesondere bei der Einschätzung der Vertrauenswürdigkeit und Neutralität digitaler Gesundheitsinformationen.

Der Beitrag von *Freye und Buchner* diskutiert rechtliche Rahmenbedingungen bei der Forschung zu und mit digitalen Gesundheitstechnologien. Der Artikel führt aus, dass individuelle Einwilligungskonzepte zu Unsicherheiten bei der Anwendung von rechtlichen Regelungen führen können und daher der Forschungsdatenschutz weiterentwickelt werden muss.

Die anschließenden 3 Artikel befassen sich mit digitalen Lebenswelten und Public Health. Einen besonderen Fokus auf Kinder und Jugendliche legt der Beitrag von *Hebestreit und Sina*. Sie erläutern, inwieweit ungesunde Ernährung mit digitaler Mediennutzung in Verbindung steht, betrachten aber auch Potenziale der Gesundheitsförderung. Die Autorinnen begründen, dass unangemessene digitale Werbung für nicht kindergerechte Lebensmittel einzuschränken ist.

Der Beitrag von *Schüz und Jones* geht auf soziale Medien und die dort häufig anzutreffenden Falsch- und Desinformationen zu Gesundheitsthemen ein. Die Analyse der Verbreitungsmuster von Falschinformationen wird zunehmend bedeutsamer, auch um gezielte Maßnahmen zur Eindämmung der Ausbreitung zu entwickeln. Der Beitrag stellt mögliche Ansatzpunkte dar, benennt jedoch auch viele offene Fragen.

Dark Patterns, also manipulative oder täuschende Designstrategien, finden sich insbesondere in nicht geprüften oder nicht regulierten digitalen Gesundheitsanwendungen. Sie sollen meist Nutzer:innen zu Entscheidungen im Sinne des Anbieters der Anwendung bewegen. Der Beitrag von *Mildner und Koautoren* gibt einen Überblick über diese Strategien und beschreibt aktuelle und zukünftige Ansätze für eine sichere Nutzung von Gesundheitsanwendungen.

Die folgenden 3 Beiträge beleuchten das Potenzial digitaler Technologien für Public-Health-Aufgaben, der abschließende Beitrag geht auf Digital Public Health in der akademischen Ausbildung ein. *Shresta und Koautorinnen* stellen digitale Anwendungen an der Schnittstelle Public Health und Stadtplanung vor, die neue Formen der Beteiligung an einer gesundheitsgerechten Stadtplanung ermöglichen. Dabei wird auch deutlich, dass digitale und analoge Planungsschritte für gelingende Partizipation klug verschränkt werden müssen.

*Wolf-Ostermann und Rothgang* stellen die Frage, was digitale Technologien in der Pflege leisten können. Das Potenzial in Hinsicht auf verbesserte Pflegequalität und Arbeitsbedingungen wird allerdings dem Beitrag zufolge noch unzureichend genutzt. So fehlen integrative Schulungs- und Finanzierungsmodelle sowie methodisch hochwertige Evaluationen.

Im Folgebeitrag beschäftigen sich *Fuhr und Koautor:innen* mit den Funktionen und der Anwendung digitaler Technologien für psychische Gesundheit. Der Artikel weist auf die große Vielfalt überwiegend ungetesteter digitaler Interventionen hin, skizziert neue Ansätze wie das digitale Phänotypisieren und beschreibt digitale Interventionsstudien im Rahmen des LWC DiPH.

Inwieweit Digital Public Health mittlerweile Eingang in die Public-Health-Ausbildung an öffentlichen Hochschulen gefunden hat, analysieren *Albrecht und Koautor:innen* im letzten Beitrag anhand von Modulhandbüchern. Nur in 16 von 79 Vollzeitstudiengängen konnten Digital-Public-Health-Elemente identifiziert werden, d. h., in der großen Mehrzahl wird der Bereich noch weitgehend ausblendet.

Zusammen bieten die Beiträge in diesem Heft einen zugegebenermaßen subjektiven, aber so auch fokussierten und beispielhaften Überblick über zentrale Befunde zu Auswirkungen, Potenzialen und Herausforderungen für Digital Public Health. Es wird deutlich, dass eine systematische wissenschaftliche Auseinandersetzung mit Digital Public Health, die über Begleitforschung hinausgeht, wichtig ist und gleichzeitig neue Fragestellungen und Paradigmen entwickelt werden müssen. Die grundlegend multidisziplinäre Ausrichtung von Public Health bietet ideale Voraussetzungen, die verschiedenen wissenschaftlichen Bereiche zu integrieren. So kann gewährleistet werden, dass ein hochrelevantes aktuelles Entwicklungsfeld in Richtung der Erfüllung zentraler Public-Health-Aufgaben kooperativ gestaltet wird, anstatt hauptsächlich als Fortschritts- und Anwendungsbereich für neue Technologien diskutiert zu werden.

Wir wünschen Ihnen viel Freude bei der Lektüre!

Hajo Zeeb, Iris Pigeot und Benjamin Schüz

